# Integrated analysis of transcriptome, metabolome, and histochemistry reveals the response mechanisms of different ages *Panax notoginseng* to root-knot nematode infection

**DOI:** 10.3389/fpls.2023.1258316

**Published:** 2023-09-14

**Authors:** Zhuhua Wang, Wenpeng Wang, Wentao Wu, Huiling Wang, Shuai Zhang, Chen Ye, Liwei Guo, Zhaoxia Wei, Hongping Huang, Yixiang Liu, Shusheng Zhu, Youyong Zhu, Yang Wang, Xiahong He

**Affiliations:** ^1^ State Key Laboratory for Conservation and Utilization of Bio-Resources in Yunnan, Yunnan Agricultural University, Kunming, Yunnan, China; ^2^ Academy of Science and Technology, Chuxiong Normal University, Chuxiong, Yunnan, China; ^3^ School of Landscape and Horticulture, Southwest Forestry University, Kunming, Yunnan, China

**Keywords:** *Panax notoginseng*, root-knot nematode, phenylpropanoid pathways, lignin, transcriptome, metabolome

## Abstract

*Panax notoginseng* (*P. notoginseng*) is an invaluable perennial medicinal herb. However, the roots of *P. notoginseng* are frequently subjected to severe damage caused by root-knot nematode (RKN) infestation. Although we have observed that *P. notoginseng* possessed adult-plant resistance (APR) against RKN disease, the defense response mechanisms against RKN disease in different age groups of *P. notoginseng* remain unexplored. We aimed to elucidate the response mechanisms of *P. notoginseng* at different stages of development to RKN infection by employing transcriptome, metabolome, and histochemistry analyses. Our findings indicated that distinct age groups of *P. notoginseng* may activate the phenylpropanoid and flavonoid biosynthesis pathways in varying ways, leading to the synthesis of phenolics, flavonoids, lignin, and anthocyanin pigments as both the response and defense mechanism against RKN attacks. Specifically, one-year-old *P. notoginseng* exhibited resistance to RKN through the upregulation of 5-O-p-coumaroylquinic acid and key genes involved in monolignol biosynthesis, such as PAL, CCR, CYP73A, CYP98A, POD, and CAD. Moreover, two-year-old *P. notoginseng* enhanced the resistance by depleting chlorogenic acid and downregulating most genes associated with monolignol biosynthesis, while concurrently increasing cyanidin and ANR in flavonoid biosynthesis. Three-year-old *P. notoginseng* reinforced its resistance by significantly increasing five phenolic acids related to monolignol biosynthesis, namely p-coumaric acid, chlorogenic acid, 1-O-sinapoyl-D-glucose, coniferyl alcohol, and ferulic acid. Notably, *P. notoginseng* can establish a lignin barrier that restricted RKN to the infection site. In summary, *P. notoginseng* exhibited a potential ability to impede the further propagation of RKN through the accumulation or depletion of the compounds relevant to resistance within the phenylpropanoid and flavonoid pathways, as well as the induction of lignification in tissue cells.

## 1 Introduction

Root-knot nematode (RKN) is a plant parasitic pest that can exert significant influence on the growth, quality, yield, and environmental stress tolerance of host plants (Borah et al., 2018). This destructive pathogen possesses distinct characteristics including a short life cycle, facile propagation, rapid reproduction rates, and an extensive range of hosts, rendering it one of the most detrimental plant pathogens (Wu et al., 2022). Currently, approximately 100 species of RKN have been documented globally, with over 5500 plant species identified as hosts, encompassing crops, vegetables, flowers, diverse tree species, spices, and even medicinal plants (Blok et al., 2010; Wang et al., 2021). Previous investigations have revealed that the medicinal plant *Panax notoginseng* frequently suffers severe damage inflicted by RKN, leading to substantial yield and quality losses (Yang et al., 2019; Wang et al., 2021).

*Panax notoginseng* [*P. notoginseng* (Burk.) F. H. Chen], a perennial herb of significant economic and medicinal value, has been cultivated for over 400 years in Yunnan province, Southwest China (Liu et al., 2019a; Wei et al., 2019). Previous studies have indicated that *P. notoginseng* has effects on antioxidative, antiaging, and anticancer, and can promote blood circulation (Wang et al., 2016). Recently, due to the escalating demand for *P. notoginseng* in the food industry and traditional medicine, extensive large-scale plantations of *P. notoginseng* have emerged throughout China (Yang et al., 2018). However, *P. notoginseng* remains vulnerable to pests, particularly RKN which can infect its roots to cause the formation of numerous root galls and severe damage. This detrimental infestation results in stunted growth, wilting, and leaf yellowing in *P. notoginseng* (Dong et al., 2013; Braun-Kiewnick et al., 2016). Previous studies have identified *Meloidogyne hapla* as a pathogenic nematode that caused RKN disease of *P. notoginseng* (Dong et al., 2013; Yang et al., 2019; Wang et al., 2021). Moreover, the nematode invasion-induced wounds serve as the entry points for other pathogens. The interactions among bacteria, viruses, fungi pathogens, and nematodes can substantially increase the damage inflicted upon *P. notoginseng*, ultimately resulting in significant yield losses. Interestingly, our earlier studies have revealed the contrast in nematode susceptibility between *P. notoginseng* seedlings which suffer severe damage and mature plants which exhibit only mild damage, which indicated the existence of adult-plant resistance (APR) against RKN disease in *P. notoginseng* (Wang et al., 2021; Wang et al., 2023). However, the molecular mechanisms underlying host pathogenesis and defense responses against RKN disease in different stages of *P. notoginseng* still remain unexplored.

In response to the invasion of various pests and pathogens, plants have evolved intricate defense mechanisms that involve the coordination of diverse signaling pathways (Xing et al., 2017; Andersen et al., 2018). The comprehension of the mechanisms underlying plant-pathogen interactions holds great potential for mitigating crop yield and quality losses, thereby benefiting agricultural production (Andersen et al., 2018). Comparative transcriptomics has emerged as a powerful tool for investigating host-pathogen interactions in recent years (Zuluaga et al., 2015). For instance, the transcriptomic profiling has been successfully proved to possess the defense mechanisms of chrysanthemum leaves against *Alternaria* infection (Liu et al., 2020a). Similarly, the transcriptome analysis has been effectively employed to unveil the mechanisms underlying nematode-host interactions during RKN infection in tobacco, alfalfa, cotton, sweet potato, etc. (Postnikova et al., 2015; Xing et al., 2017; Kumar et al., 2019). Moreover, the roles of sweet potato peroxidase genes in protecting plants against RKN infection have been identified through transcriptomic approaches (Sung et al., 2019). Notably, a transcriptome analysis has indicated the potential of jasmonate and ethylene signaling pathways in conferring increased resistance to *Fusarium solani* in *P. notoginseng* (Liu et al., 2019b). In addition, a previous study based on the transcriptome analysis uncovered the role of 2,3-butanediol in activating resistance against leaf disease infection in *P. notoginseng* (Li et al., 2022). However, no published study has delved into the gene expression profiling in *P. notoginseng* infected with RKN, leaving this aspect unexplored.

Metabolomics, a rapidly advancing technology, plays a vital role in elucidating the intricate growth processes of plants and their complex responses to various abiotic and biotic stresses through the identification of diverse compounds (Shulaev et al., 2008). It has exhibited the extensive applications in studying plant stress responses (Nakabayashi and Saito, 2015). For example, comparative metabolomics analyses have successfully revealed the key metabolites involved in alkali or salinity stress responses in crops such as safflower (Zhao et al., 2015), alfalfa (Song et al., 2017), peanut (Cui et al., 2018), and barley (Wang et al., 2019). In recent years, numerous researchers have employed combined transcriptomics and metabolomics approaches to uncover mechanisms underlying plant-pathogen and plant-insect pest interactions (Coppola et al., 2019; Chen et al., 2020). Notably, such integrated methodologies have been utilized to investigate the response mechanisms of *P. notoginseng* to abiotic stress, such as the decrease in flavonoids and related genes following inflorescence removal (Bai et al., 2021). However, our current understanding of the defense mechanisms employed by *P. notoginseng* plants remains limited, and the mechanisms underlying *P. notoginseng*’s response to RKN stress at different stages of plant development are not yet fully elucidated.

In this study, we aimed to elucidate the response mechanisms of *P. notoginseng* at different stages of development to RKN infection by employing transcriptome, metabolome, and histochemical analyses. To achieve this, we identified differentially expressed genes (DEGs) and differentially accumulated metabolites (DAMs) between the healthy and diseased *P. notoginseng* plants at each age group. Subsequently, we performed concurrent mapping of the DAMs and DEGs onto the Kyoto Encyclopedia of Genes and Genomes (KEGG) pathway, allowing to determine the significantly enriched pathways. Through the detailed investigation of the gene and metabolite changes within these important pathways, we unveiled the intricate mechanisms governing the response of *P. notoginseng* at different ages to RKN infection. Furthermore, we conducted histochemical analyses to explore the accumulation of lignin in fibrous roots of *P. notoginseng* plants at different ages. The response mechanisms of *P. notoginseng* at different ages to RKN infection were analyzed at morphological, metabolic and molecular levels. The results may provide new insights into the transcriptional and metabolic regulation patterns of *P. notoginseng* at different ages during RKN infection. Moreover, these insights would contribute to the development of novel strategies and resources for sustainable and ecologically sound management of RKN disease.

## 2 Materials and methods

### 2.1 Plant materials

A greenhouse pot experiment was conducted at the experimental station of Yunnan Agricultural University in Xundian County, Yunnan, China (103^°^15′ E, 25^°^30′ N; altitude of 1,866 m) to investigate the response mechanisms of *P. notoginseng* at different ages to RKN damage. On January 1, 2020, healthy *P. notoginseng* seeds, seedlings, and two-year-old seedlings were planted in plastic container pots (15 cm × 12.8 cm × 10 cm) containing nematode-infested soil. There was an average of 20 second-stage juveniles (J2s) per 100 g of background soil, and each pot contained 1500 g of soil. As a result, there were approximately 300 J2s per pot. Each replicate consisted of 100 pots, and three replicates were established for each age group of *P. notoginseng*. On September 16, 2020, samples of *P. notoginseng* from each age group were collected, and the disease incidence and disease index were calculated (Yang et al., 2014). Subsequently, the healthy and diseased samples were separated. Each group consisted of 30 plants, with triplicate samples. The one-year-old, two-year-old, and three-year-old *P. notoginseng* samples were designated as H1-1, 2, 3 (healthy), D1-1, 2, 3 (diseased), H2-1, 2, 3 (healthy), D2-1, 2, 3 (diseased), H3-1, 2, 3 (healthy), and D3-1, 2, 3 (diseased). The samples were carefully washed with water and surface disinfected with 75% alcohol (Xin M., 2021). Taproots were removed, and 5 g of fibrous roots were collected from each group for further analysis. The samples were immediately positioned in liquid nitrogen, stored at −80°C for subsequent metabolomics, transcriptomics, and real-time qPCR analysis.

### 2.2 RNA extraction, sequencing, RNA-seq data analysis, annotation, and analysis of DEGs

RNA extraction, quality and quantity detection, library construction, and sequencing were performed by Metware Biotechnology Co. Ltd. (Wuhan, China) through utilizing the Illumina HiSeq4000 platform (http://www.metware.cn/). The original sequenced data underwent a series of preprocessing steps, including adapter sequence trimming, removal of ambiguous bases, and elimination of low-quality reads, to obtain a set of high-quality standard reads. All clean reads that passed the filtering criteria were mapped to the reference genome of *P. notoginseng* (Yang et al., 2021) via HISAT2 (http://ccb.jhu.edu/software/hisat2/index.shtml). This alignment process allowed for the determination of the precise genomic or gene locations of the reads, as well as the unique sequence characteristics specific to each sample, which were essential for the subsequent analysis (Kim et al., 2015). The expression levels of genes/transcripts in individual reads were then normalized to FPKM (Trapnell et al., 2010). Pearson’s correlation coefficient was employed to evaluate the correlation between biological samples, with a minimum R^2^ value of 0.8 considered indicative of a strong correlation among biological replicates. Principal component analysis (PCA) was performed on the gene/transcript expression values to gain insights into the overall differences in gene/transcript profiles across samples (Zhao et al., 2021).

In this study, DEGs were identified between the healthy and diseased groups based on the selection criteria of |log2 Fold Change| >= 1 and false discovery rate (FDR) < 0.05 (Liu et al., 2020b; Shi et al., 2021). The total number of DEGs, as well as the number of upregulated and downregulated genes, were determined for each comparison between different samples, and all DEGs were included in subsequent analyses. The Venn diagram was generated to visualize the overlap or uniqueness of DEGs among different comparison combinations. Subsequently, the unigenes were comprehensively annotated, and pathway enrichment analyses were conducted through applying the KEGG (Liu et al., 2020a; Shi et al., 2021).

### 2.3 RT-qPCR verification

The usability of the RNA-Seq data and the results of the differential expression analysis were validated through the application of quantitative real-time reverse-transcription PCR (qRT-PCR) technology (Mei et al., 2020; Xin et al., 2021). Twelve disease-resistant-related DEGs from the phenylpropanoid biosynthesis pathway were specifically selected for qRT-PCR verification. Gene-specific primer pairs for these twelve DEGs and 18S rRNA gene were in **Supplementary Table 1**. The qRT-PCR was performed through applying a 2X SG Fast qPCR Master Mix kit (High Rox, B639273, BBI, ABI) according to the standard protocol. The expression levels of the genes were measured with a Thermo Fisher QuantStudio 3 and 5 Real-Time PCR system (ABI/Thermo Fisher, USA).

### 2.4 Metabolite extraction and LC-MS/MS analysis

Metabolite extraction, identification, and quantitative analysis were performed using a widely targeted metabolome method by Wuhan Metware Biotechnology Co., Ltd. (Wuhan, China). After taproots removal, 18 freeze-dried fibrous root samples of *P. notoginseng* were ground to a fine powder (Peng et al., 2022). Metabolites were then extracted following the reagents and methods outlined by Chen et al. (2013). All the sample extracts were analyzed via an ultraperformance liquid chromatography (UPLC) system (SHIMADZU Nexera X2). The UPLC system was equipped with an Agilent SB-C18 column (1.8 μm, 2.1 mm × 100 mm) with a flow rate of 0.35 mL/min. The mobile phase consisted of solvent A, pure water (0.1% formic acid) and solvent B, acetonitrile (0.1% formic acid). The elution gradients were as follows: from 0.00 to 9.00 min, 95% A decreased linearly to 5%, simultaneously coupled with a linear increase from 5% B to 95% B. This composition of 5% A and 95% B was kept for 1 min. Subsequently, within the interval of 10.00 to 11.10 min, 5% A increased linearly to 95%, alongside a linear decrease from 95% B to 5% B A composition of 95% A and 5% B was equilibrated to 14 min. The column temperature was maintained at 40°C, and the injection volume was 4 μL (Peng et al., 2022).

### 2.5 Identification and quantitative analysis of metabolites

Utilizing the self-built database of Wuhan MetWare Biological Science and Technology Co., Ltd., qualitative analysis of metabolites was performed based on the primary and secondary MS data information (Bai et al., 2021). For the quantitative analysis of metabolites, a scheduled multiple reaction monitoring model was used (Chen et al., 2013). Pearson’s correlation coefficient was utilized to evaluate the correlation between biological samples, with a higher R^2^ indicating a stronger correlation. The identified metabolites from the 18 samples of *P. notoginseng* were subjected to PCA, and the resulting top two principal components (PC1 and PC2) were visualized through utilizing two-dimensional scatter plots (Li et al., 2022).

The criteria of significant DAMs screening were as follows: fold change ≥ 2 and fold change ≤ 0.5, along with variable importance in projection (VIP) ≥ 1. The total number of DAMs, as well as the up-regulated and down-regulated number of DAMs between the healthy and diseased groups, were determined, and all DAMs were included in subsequent analyses. A Venn diagram was generated to visualize the overlap of DAMs among different comparison combinations. To facilitate analysis, all significant DAMs were normalized using unit variance scaling, and a cluster analysis heatmap of the samples was constructed through utilizing the R software heatmap package. The identified metabolites were annotated applying the KEGG compound database (http://www.kegg.jp/kegg/compound/), followed by the mapping of the annotated metabolites to their corresponding pathways using the KEGG pathway database (http://www.kegg.jp/kegg/pathway.html) (Xia et al., 2021).

### 2.6 Co-joint analysis of the transcriptome and metabolome

By combining the results of the metabolomics-related DAMs analysis and the transcriptomics-related DEG analysis, the DAMs and DEGs belonging to the same groups were simultaneously mapped onto the KEGG pathway. The enrichment results of the DAMs and DEGs were utilized to generate a P-value histogram, which illustrated the degree of pathway enrichment achieved by both sets of data simultaneously.

### 2.7 Histochemistry analysis of lignin morphology and distribution

The fresh healthy fibrous roots of *P. notoginseng* were cut into 0.5-1.0 cm lengths, and the diseased root knots were removed. Subsequently, the samples were vacuum-fixed in FAA solution (90 mL of 70% ethanol, 5 mL of 40% formaldehyde, and 5 mL of glacial acetic acid) at room temperature for a minimum of 48 h (Zhou et al., 2021). The tissues were then dehydrated via a series of graded ethanol concentrations, with each step lasting 2 h. Following the dehydration, the transparency processing of the dehydrated tissues was conducted in a mixture of ethanol and xylene for 30 min with ratios of 2:1, 1:1, and 1:2, followed by two changes of a 100% xylene solution. Subsequently, the obtained samples were embedded in wax (Zhou et al., 2021). For sectioning, a sliding microtome (Leica RM2016, Wetzlar, Germany) was used to obtain slices measuring 10–12 mm in thickness. The paraffin slides were dewaxed and stained with phloroglucinol dye for 2 min, and images were captured via an imaging system (Nikon DS-U3, Nikon) and saved within 3 min. The lignified components appeared red, the background remained colorless, or the tissue itself exhibited distinct colors.

### 2.8 Statistical analysis

All the data analyses were performed using SPSS 19.0 software (SPSS Inc., Chicago, IL, United States). The statistical analysis was conducted using One-way ANOVA followed by the Tukey test, with a significance level set at p < 0.05 (Liu et al., 2020c).

## 3 Results

### 3.1 Disease incidence and disease index of RKN disease in different ages *P. notoginseng*

**Figure 1:**
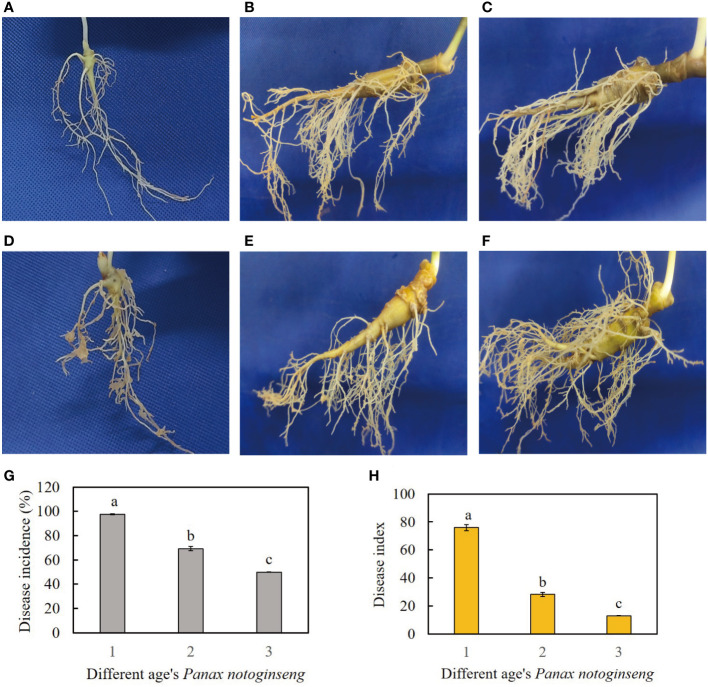
**(A–C)** represent healthy samples of one-year-old, two-year-old, and three-year-old *P. notoginseng*, respectively. **(D–F)** represent diseased samples of one-year-old, two-year-old, and three-year-old *P. notoginseng*, respectively. **(G)** Disease incidence of RKN disease in different age’s *P. notoginseng*. **(H)** Disease index of RKN disease in different age’s *P. notoginseng*. The different lowercase letters (a, b) above the error bars indicate significant differences among different age’s *P. notoginseng* (Duncan, p < 0.05).

The field data collected in 2020 revealed varying levels of disease severity in different age groups of *P. notoginseng* resulting from RKN infestation, with the one-year-old *P. notoginseng* exhibiting the highest severity (**Figure 1**). RKN primarily infected the lateral roots and fibrous roots of *P. notoginseng*. In addition, RKN formed numerous evident galls in the roots of one-year-old *P. notoginseng*, while sporadic small galls were only observed in the roots of two or three-year-old *P. notoginseng* (**Figures 1D–F**). The disease incidence of RKN infection in one-year-old *P. notoginseng* was 97.6%, significantly higher than that in two-year-old *P. notoginseng* (69.3%) and three-year-old *P. notoginseng* (49.8%), showing a significant downward pattern (**Figure 1G**). Moreover, the disease index of RKN infection in one-year-old *P. notoginseng* was 76, significantly higher than that in two-year-old *P. notoginseng* (28.2) and three-year-old *P. notoginseng* (12.9), also exhibiting a significant downward trend (**Figure 1H**).

### 3.2 Preliminary analysis of transcriptome data of the healthy/diseased *P. notoginseng*

To investigate the transcriptomic alterations and underlying regulatory mechanisms in the fibrous roots of *P. notoginseng* at different ages in response to nematode infestation, RNA sequencing was conducted on both healthy and diseased samples from each age group. A total of 18 sample libraries were constructed and subjected to high-throughput sequencing, resulting in the generation of 120.99 Gb of high-quality clean reads. Each individual sample yielded approximately 6 Gb of clean reads (**Supplementary Table 2**). The quality assessment of the *P. notoginseng* fibrous root samples indicated Q20 values ranging from 97.46% to 98.21%, and Q30 values ranging from 92.49% to 94.55% across different age groups (**Supplementary Table 2**). The GC contents ranged from 42.96% to 43.29% for each sample (**Supplementary Table 2**). Subsequently, the match ratios ranged from 90.37% to 93.85%, with unique mapped ratios ranging from 82.11% to 85.56% (**Supplementary Table 3**).

**Figure 2:**
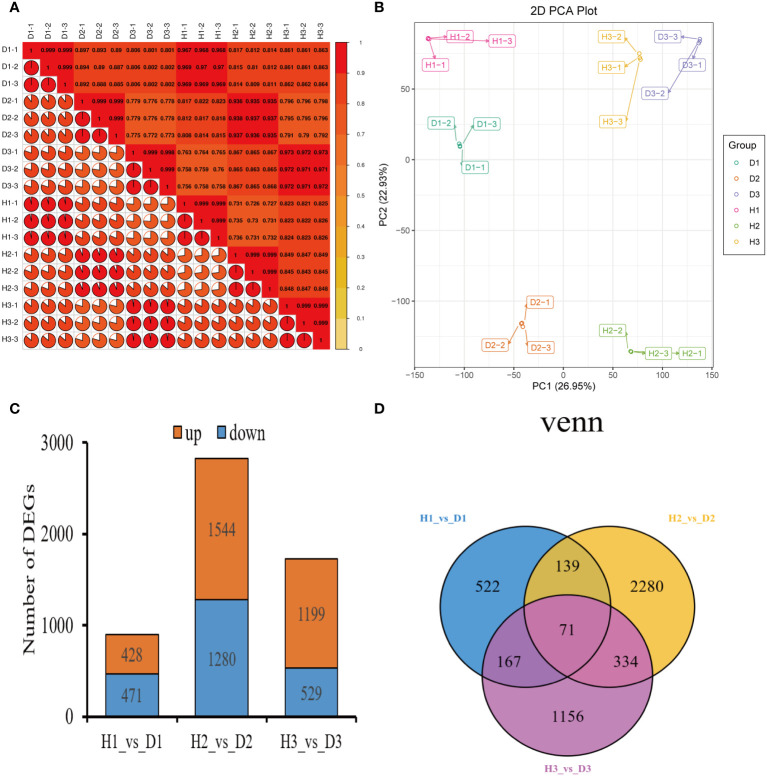
Preliminary analysis of *P. notoginseng* transcriptomic data and comparative analysis of DEGs. **(A)** Pearson’s correlation heat map of different age’s healthy/diseased *P. notoginseng* samples. **(B)** PCA score plot of different age’s healthy/diseased *P. notoginseng* samples. **(C)** Total number of up-regulated and down regulated DEGs. **(D)** Venn diagram of DEGs among the H1 vs. D1, H2 vs. D2, and H3 vs. D3 comparison groups.

The Pearson’s correlation coefficient, employed to assess the relationship among the three biological replicates of both healthy and diseased *P. notoginseng* samples across different ages, exhibited a remarkably high value surpassing 0.998, thereby indicating a robust correlation (**Figure 2A**). Specifically, when evaluating the correlation between the H1 and D1 samples, the Pearson’s correlation coefficient ranged from 0.968 to 0.97 (**Figure 2A**). Similarly, the correlation between the H2 and D2 samples ranged from 0.935 to 0.938 (**Figure 2A**), while the correlation between the H3 and D3 samples ranged from 0.971 to 0.973 (**Figure 2A**). In stark contrast, different age groups of healthy and diseased *P. notoginseng* samples displayed a feeble correlation (**Figure 2A**). The PCA indicated a discernible trend of segregation existed between the diseased samples and the healthy samples within each age group, indicating substantial differences in the gene expression profiles (**Figure 2B**).

### 3.3 Identification of DEGs in the healthy/diseased *P. notoginseng*

To investigate the disparities in gene expression between healthy and diseased *P. notoginseng* samples for each age groups, three pairwise comparisons were established: H1 vs. D1, H2 vs. D2, and H3 vs. D3. A total of 899 DEGs (428 up-regulated and 471 down-regulated) were identified in the H1 vs. D1 comparison. Similarly, the H2 vs. D2 comparison revealed 2824 DEGs (1544 up-regulated and 1280 down-regulated), while the H3 vs. D3 comparison yielded 1728 DEGs (1199 up-regulated and 529 down-regulated) (**Figure 2C**). The Venn diagram distribution demonstrated that 4669 DEGs were obtained among the three comparison groups (**Figure 2D**), with only 71 DEGs overlapping among the H1 vs. D1, H2 vs. D2, and H3 vs. D3 comparisons (**Figure 2D**). Moreover, 522, 2280, and 1156 DEGs exhibited specific expression patterns in the H1 vs. D1, H2 vs. D2, and H3 vs. D3 comparisons, respectively (**Figure 2D**). These findings indicated the differential expression of genes in healthy and diseased *P. notoginseng* samples across distinct age groups.

### 3.4 KEGG enrichment analysis of DEGs of the healthy/diseased *P. notoginseng*

**Figure 3:**
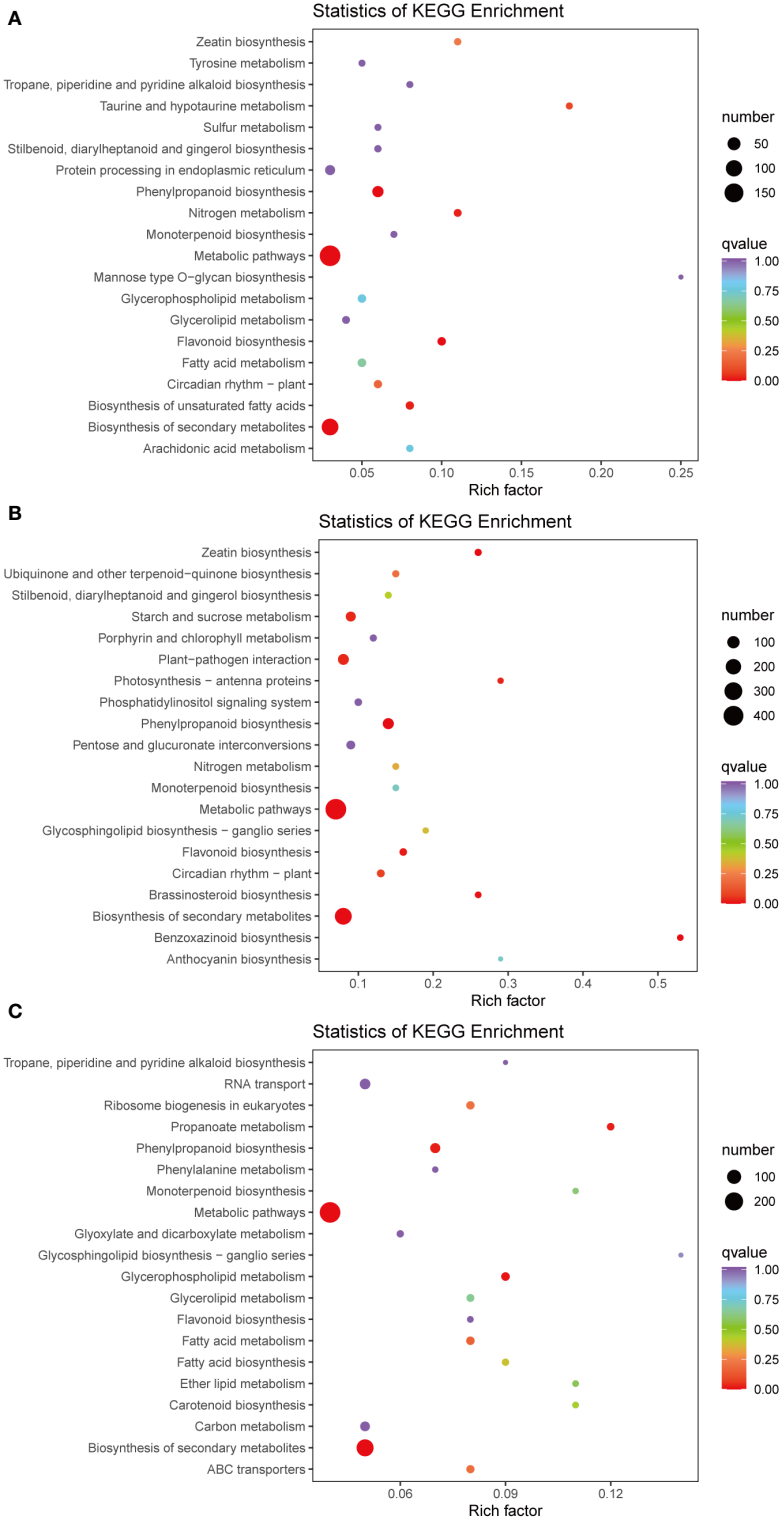
Pathway enrichment analysis of DEGs of H1 vs. D1 **(A)**, H2 vs. D2 **(B)**, and H3 vs. D3 **(C)**. The color of the point represents p-value, and the size of the point represents the number of differentially enriched metabolites.

The KEGG pathway enrichment analysis revealed the presence of 113, 126, and 125 enriched terms in the H1 vs. D1, H2 vs. D2, and H3 vs. D3 comparison groups, respectively. In the H1 vs. D1 comparison, the top five pathways identified were metabolic pathways (ko01100), phenylpropanoid biosynthesis (ko00940), biosynthesis of secondary metabolites (ko01110), flavonoid biosynthesis (ko00941), and biosynthesis of unsaturated fatty acids (ko01040) (**Figure 3A**). Similarly, in the H2 vs. D2 comparison, the top five terms comprised phenylpropanoid biosynthesis (ko00940), metabolic pathways (ko01100), biosynthesis of secondary metabolites (ko01110), benzoxazinoid biosynthesis (ko00402), and zeatin biosynthesis (ko00908) (**Figure 3B**). Furthermore, in the H3 vs. D3 comparison, the top five pathways were biosynthesis of secondary metabolites (ko01110), metabolic pathways (ko01100), glycerophospholipid metabolism (ko00564), phenylpropanoid biosynthesis (ko00940), and propanoate metabolism (ko00640) (**Figure 3C**).

### 3.5 Verification of RNA-seq data via qRT-PCR

**Figure 4:**
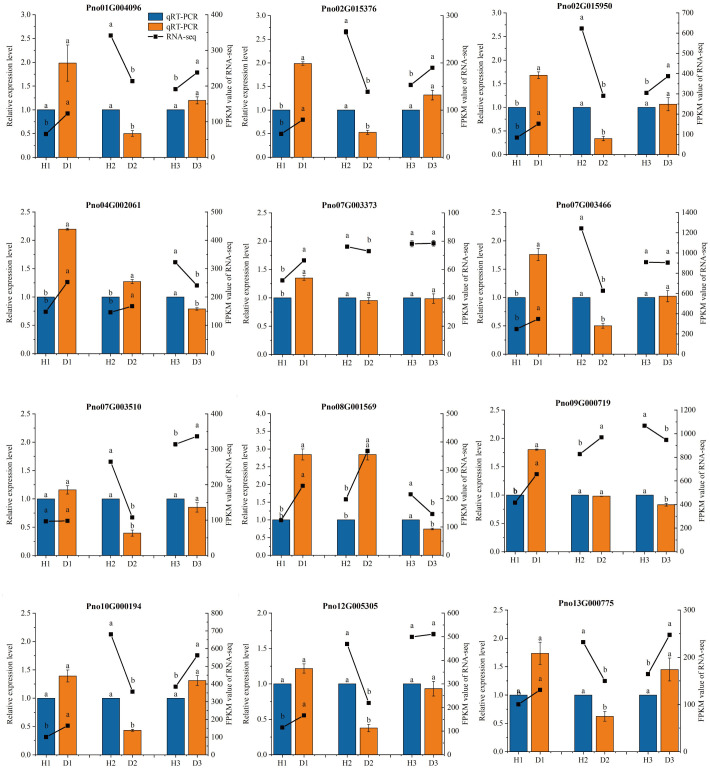
qRT-PCR validation of gene expression levels in the transcriptome. The different lowercase letters (a, b) above the error bars indicate significant differences between the healthy and diseased P. notoginseng plants at each age group (Duncan, p < 0.05).

The qRT-PCR results exhibited a consistent expression pattern with the RNA-seq results (**Figure 4**), confirming the credibility and reproducibility of the RNA-seq data. Further analysis revealed distinct expression patterns of the selected genes in diseased *P. notoginseng* at different ages compared to their healthy counterparts. In one-year-old *P. notoginseng*, RKN infection significantly up-regulated the expression of 11 genes, including peroxidase (POD) (Pno04G002061) (Pno08G001569), 5-O-(4-coumaroyl)-D-quinate 3’-monooxygenase (Pno01G004096), beta-glucosidase (Pno04G002061) (Pno08G001569), caffeoyl-CoA O-methyltransferase (Pno12G005303), shikimate O-hydroxycinnamoyltransferase (Pno02G015376), trans-cinnamate 4-monooxygenase (Pno10G000194), 4-coumarate–CoA ligase (Pno02G015950), and cinnamyl-alcohol dehydrogenase (CAD) (Pno07G003466) (Pno09G000719) (**Figure 4**). Similarly, in two-year-old *P. notoginseng*, RKN infection significantly up-regulate the expression of POD (Pno04G002061) (Pno08G001569) and CAD (Pno09G000719) (**Figure 4**). However, in three-year-old *P. notoginseng*, RKN infection significantly down-regulated the expression of POD (Pno04G002061) (Pno08G001569) and CAD (Pno09G000719) when compared to their healthy counterparts (**Figure 4**).

### 3.6 Preliminary analysis of metabolomics data of the healthy/diseased *P. notoginseng*

**Figure 5:**
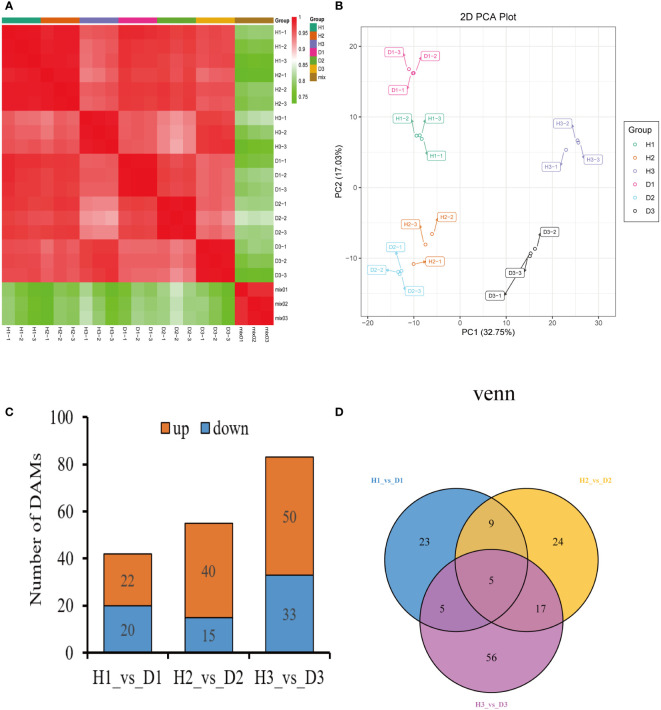
Preliminary analysis of metabolomics data of *P. notoginseng* samples. **(A)** Heatmap of sample-to-sample correlation analysis. **(B)** PCA score plot of different age’s healthy/diseased *P. notoginseng* samples. **(C)** Total number of up-regulated and down regulated DAMs. **(D)** Venn diagram of DAMs among the H1 vs. D1, H2 vs. D2, and H3 vs. D3 comparison groups.

To investigate the metabolic mechanisms underlying nematode infection in *P. notoginseng* fibrous roots at different ages, comprehensive profiling of metabolites was performed in both healthy and diseased samples. A total of 687 metabolites were identified across all samples, encompassing 116 flavonoids, 103 lipids, 91 phenolic acids, 82 amino acids and derivatives, 59 organic acids, 56 nucleotides and derivatives, 47 terpenoids, 46 alkaloids, 13 lignans and coumarins, 5 tannins, and 69 others (**Supplementary Table 4**). The correlation analysis, visualized through a heatmap, revealed a strong positive correlation among the three biological replicates of each age’s healthy and diseased *P. notoginseng* samples (**Figure 5A**). This observation indicated excellent repeatability among the replicates. Furthermore, the PCA diagram demonstrated clear differences in metabolite profiles between diseased and healthy *P. notoginseng* samples at the same age (**Figure 5B**). These findings indicated the distinct metabolic profiles and accumulation patterns between diseased and healthy *P. notoginseng* samples of the same age.

### 3.7 Identification of DAMs in the healthy/diseased *P. notoginseng*

To compare the metabolite abundance between healthy and diseased *P. notoginseng* samples at each age, three pairwise comparisons were established: H1 vs. D1, H2 vs. D2, and H3 vs. D3. In the H1 vs. D1 comparison, a total of 42 DAMs were identified, with 22 DAMs up-regulated and 20 DAMs down-regulated (**Figure 5C**). Similarly, the H2 vs. D2 comparison revealed 55 DAMs, including 40 up-regulated and 15 down-regulated DAMs (**Figure 5C**). Additionally, the H3 vs. D3 comparison yielded 83 DAMs, with 50 up-regulated and 33 down-regulated DAMs (**Figure 5C**). The Venn diagram analysis demonstrated the presence of 139 DAMs shared among the three comparison groups (**Figure 5D**) with only 5 DAMs overlapping in the H1 vs. D1, H2 vs. D2, and H3 vs. D3 comparisons (**Figure 5D**). Moreover, 23, 24, and 56 metabolites exhibited specific abundance in the H1 vs. D1, H2 vs. D2, and H3 vs. D3 comparisons, respectively (**Figure 5D**).

### 3.8 Classification and KEGG enrichment analysis of DAMs of healthy/diseased *P. notoginseng*

**Figure 6:**
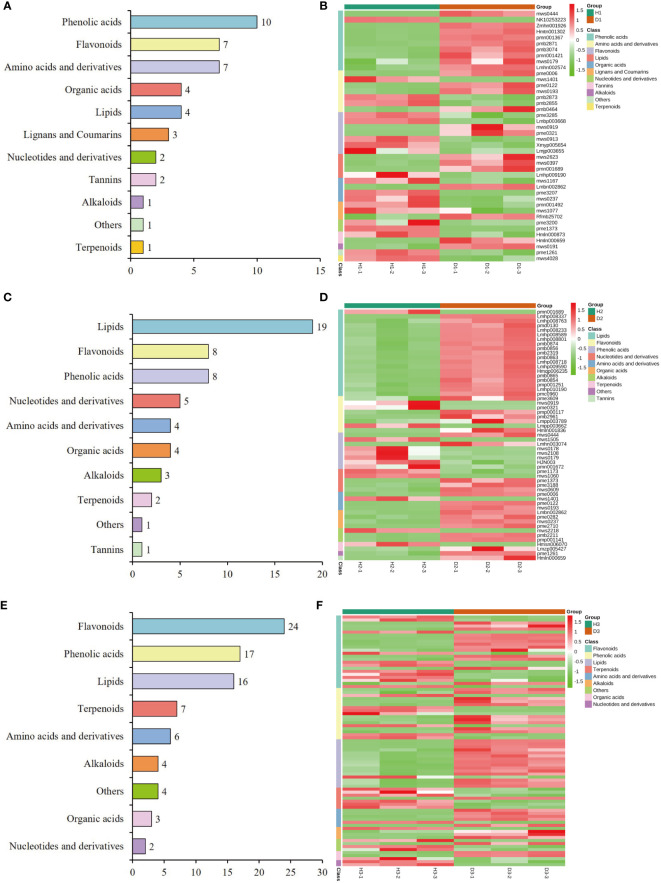
Classification and abundance of the DAMs of healthy/diseased *P. notoginseng* at different ages. Classification results of the DAMs of H1 vs. D1 **(A)**, H2 vs. D2 **(C)**, and H3 vs. D3 **(E)**. Abundance level of the DAMs of H1 vs. D1 **(B)**, H2 vs. D2 **(D)**, and H3 vs. D3 **(F)**.

To gain further insights into the profiles of DAMs, hierarchical clustering analysis was employed to classify and assess the relative abundances of these DAMs among healthy and diseased *P. notoginseng* samples at different ages. In the H1 vs. D1 comparison, cluster analysis categorized the 42 DAMs into 11 distinct groups, including 10 phenolic acids, 7 flavonoids, 7 amino acids and derivatives, 4 organic acids, 4 lipids, 3 lignans and coumarins, 2 tannins, 2 nucleotides and derivatives, 1 terpenoid, 1 alkaloid, and 1 other (**Figure 6A**, **Supplementary Table 5**). The heatmap revealed that the levels of most phenolic acids, lipids, and alkaloids were increased in diseased one-year-old *P. notoginseng* (D1) compared to healthy *P. notoginseng* (H1) (**Figure 6B**, **Supplementary Table 5**). Additionally, the KEGG enrichment analysis indicated that the DAMs identified in the H1 vs. D1 comparison were primarily associated with flavone and flavonol biosynthesis (ko00944), flavonoid biosynthesis (ko00941), and phenylpropanoid biosynthesis (ko00940) (**Supplementary Figure 1A**).

The classification analysis revealed that the 55 DAMs identified in the H2 vs. D2 comparison was attributed to 10 distinct categories, including 19 lipids, 8 flavonoids, 8 phenolic acids, 5 nucleotides and derivatives, 4 organic acids, 4 amino acids and derivatives, 3 alkaloids, 2 terpenoids, 1 tannins, and 1 other (**Figure 6C**, **Supplementary Table 6**). Among these categories, lipids, organic acids, and amino acids and derivatives indicated significantly higher levels in diseased two-year-old *P. notoginseng* (D2) compared to healthy *P. notoginseng* (H2) (**Figure 6D**, **Supplementary Table 6**). However, the abundance of phenolic acids in diseased two-year-old *P. notoginseng* (D2) was significantly decreased compared with healthy *P. notoginseng* (H2) (**Figure 6D**, **Supplementary Table 6**). Furthermore, the KEGG enrichment analysis indicated that the DAMs identified in the H2 vs. D2 comparison were primarily enriched in flavonoid biosynthesis (ko00941), anthocyanin biosynthesis (ko00942), phenylpropanoid biosynthesis (ko00940), and pyrimidine metabolism (ko00240) (**Supplementary Figure 1B**).

**Figure 6E** illustrates the clustering of the 83 DAMs identified in the H3 vs. D3 comparison into 9 distinct categories, namely 24 flavonoids, 17 phenolic acids, 16 lipids, 7 terpenoids, 6 amino acids and derivatives, 4 alkaloids, 3 organic acids, 2 nucleotides and derivatives, and 4 others (**Figure 6E**, **Supplementary Table 7**). The heatmap analysis revealed that the levels of most lipids, amino acids and derivatives, and alkaloids were significantly higher in three-year-old diseased *P. notoginseng* (D3) in comparison to healthy *P. notoginseng* (H3). Conversely, most terpenoids exhibited significant down-regulation in D3 compared to H3 (**Figure 6F**, **Supplementary Table 7**). Furthermore, the KEGG pathway analysis indicated that the main pathways enriched in the H3 vs. D3 comparison included phenylpropanoid biosynthesis (ko00940), flavonoid biosynthesis (ko00941), anthocyanin biosynthesis (ko00942), flavone and flavonol biosynthesis (ko00944), and phenylalanine metabolism (ko00360) (**Supplementary Figure 1C**). Overall, lipids indicated an increasing trend in each diseased *P. notoginseng* sample (D1, D2, D3) compared to each healthy *P. notoginseng* (H1, H2, H3), while phenylpropanoid biosynthesis (ko00940) and flavonoid biosynthesis (ko00941) were prominent pathways shared among the three comparison groups.

### 3.9 Co-joint Analysis of the transcriptome and metabolome

The analysis of DAMs and DEGs in the H1 vs. D1 comparison revealed that they were associated with 24 metabolic pathways. However, none of these pathways indicated significant enrichment simultaneously (**Supplementary Figure 2A**). Among the DEGs in H1 vs. D1 comparison, the most significantly enriched pathways included metabolic pathways (189 DEGs) (ko01100), biosynthesis of secondary metabolites (107 DEGs) (ko01110), phenylpropanoid biosynthesis (31 DEGs) (ko00940), flavonoid biosynthesis (10 DEGs) (ko00941), and biosynthesis of unsaturated fatty acids (9 DEGs) (ko01040) (p < 0.01) (**Supplementary Figure 2A**). In the H2 vs. D2 comparison, the DAMs and DEGs were allocated to 16 metabolic pathways, with only anthocyanin biosynthesis (ko00942) showing the most significant co-enrichment involving 3 DAMs and 4 DEGs (p < 0.01) (**Supplementary Figure 2B**). Among the DEGs in this comparison, the most significantly enriched pathways included metabolic pathways (447 DEGs) (ko01100), biosynthesis of secondary metabolites (255 DEGs) (ko01110), phenylpropanoid biosynthesis (69 DEGs) (ko00940), flavonoid biosynthesis (16 DEGs) (ko00941), and stilbenoid, diarylheptanoid, and gingerol biosynthesis (11 DEGs) (ko00945) (p < 0.01) (**Supplementary Figure 2B**). In the H3 vs. D3 comparison, the DAMs and DEGs were assigned to 34 metabolic pathways, with phenylpropanoid biosynthesis (ko00940) being the most significantly co-enriched pathway involving 6 DAMs and 34 DEGs (p<0.01) (**Supplementary Figure 2C**). Furthermore, metabolic pathways (281 DEGs) (ko01100), biosynthesis of secondary metabolites (169 DEGs) (ko01110), and ABC transporters (16 DEGs) (ko02010) were also among the most significantly enriched pathways for DEGs in H3 vs. D3 (p < 0.01) (**Supplementary Figure 2C**).

### 3.10 Metabolic profiling of phenylpropanoid biosynthesis pathway in the roots of *P. notoginseng* under RKN infection

**Figure 7:**
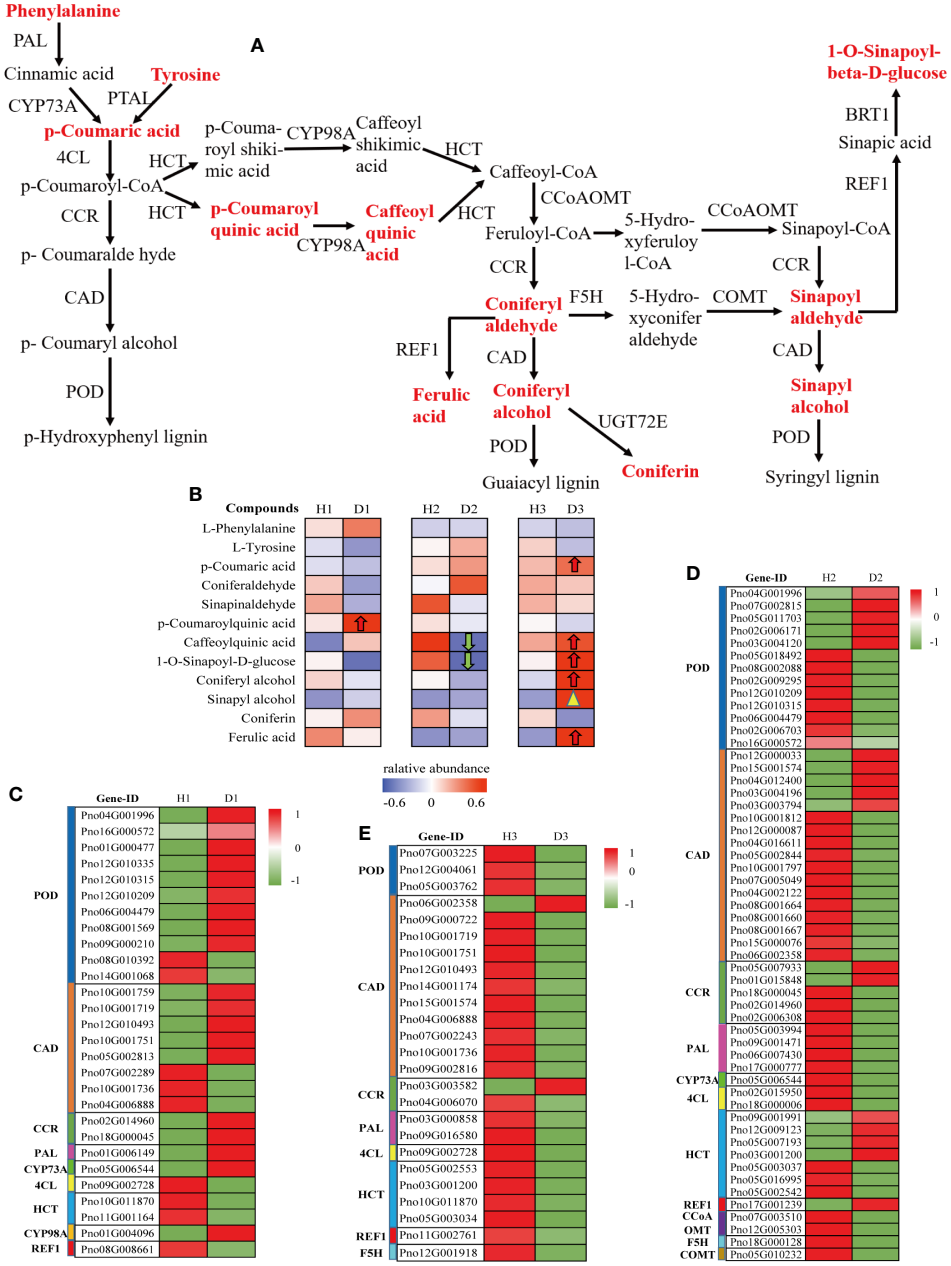
Metabolic profiling of the phenylpropanoid biosynthesis pathway in the roots of *P. notoginseng* under RKN infection. **(A)** The key pathway of monolignol biosynthesis. **(B)** Heatmap analysis of the relative abundance of detected metabolite in monolignol biosynthesis. Monolignol biosynthesis-related DEGs in H1 vs. D1 **(C)**, H2 vs. D2 **(D)**, and H3 vs. D3 **(E)**.

To gain further insights into the regulation of phenylpropanoid biosynthesis in *P. notoginseng* roots under RKN infection, we investigated the changes in both DEGs and detected metabolites associated with this pathway (ko00940). These components were simultaneously mapped onto the monolignol biosynthesis pathway (M00039), a critical branch of phenylpropanoid biosynthesis. The analysis revealed that a substantial number of DEGs and 12 metabolites in the *P. notoginseng* roots (H1 vs. D1, H2 vs. D2, H3 vs. D3) were assigned to the monolignol biosynthesis pathway (**Figure 7A**). Among the 12 detected metabolites, 5 compounds exhibited an increasing trend in expression. However, only one compound, 5-O-p-Coumaroylquinic acid (C12208), demonstrated significant up-regulation in H1 vs. D1 (**Figure 7B**). Conversely, chlorogenic acid (3-O-caffeoylquinic acid)* (C00852) and 1-O-sinapoyl-D-glucose (C01175) were significantly down-regulated in D2 compared to H2 (**Figure 7B**). In addition, in the H3 vs. D3 comparison, five compounds, namely p-coumaric acid (C00811), chlorogenic acid (3-O-caffeoylquinic acid)* (C00852), 1-O-sinapoyl-D-glucose (C01175), coniferyl alcohol (C00590), and ferulic acid (C01494), were significantly up-regulated in D3 relative to H3 (**Figure 7B**). Additionally, sinapyl alcohol (C02325) in H3 vs. D3 exhibited a notable increase close to the significant level (**Figure 7B**). These results indicated that the compounds associated with monolignol biosynthesis were predominantly upregulated in three-year-old *P. notoginseng* under RKN infection, suggesting that three-year-old *P. notoginseng* may possess a stronger resistance ability against pathogenic nematode invasion.

In this study, the heatmap analysis revealed that several DEGs were involved in the regulation of key enzymes within the monolignol biosynthesis pathway. In the H1 vs. D1 comparison, these DEGs exhibited significant up-regulation (**Figure 7C**). However, in the H2 vs. D2 and H3 vs. D3 comparisons, most DEGs presented significant downregulation (**Figure 7D, E**). Specifically, the expression levels of phenylalanine ammonia-lyase (PAL), cinnamoyl-CoA reductase (CCR), trans-cinnamate 4-monooxygenase (CYP73A), and 5-O-(4-coumaroyl)-D-quinate 3’-monooxygenase (CYP98A) genes were significantly up-regulated in one-year-old diseased *P. notoginseng* compared to one-year-old healthy *P. notoginseng*. Conversely, the 4-coumarate-CoA ligase (4CL), shikimate O-hydroxycinnamoyltransferase (HCT), and coniferyl-aldehyde dehydrogenase (REF1) genes were significantly downregulated (**Figure 7C**). The expression levels of POD and CAD indicated a general trend for gene upregulation than downregulation in H1 vs. D1 (**Figure 7C**). These results suggested that the defense system, particularly phenylpropanoid metabolism, was activated in one-year-old *P. notoginseng* under RKN infection, potentially enhancing its resistance to pathogenic nematodes. However, in the H2 vs. D2 comparison, only the REF1 gene showed significant up-regulation, while the PAL, 4CL, CYP73A, caffeoyl-CoA O-methyltransferase (CCoAOMT), ferulate-5-hydroxylase (F5H), and caffeic acid 3-O-methyltransferase (COMT) genes were significantly down-regulated (**Figure 7D**). Moreover, compared to H2, the expression of POD, CAD and CCR involved in D2 showed a higher number of down-regulated genes than up-regulated genes (**Figure 7D**). Interestingly, in the H3 vs. D3 comparison, the expression levels of POD, CAD (except Pno06G002358), CCR (except Pno03G003582), PAL, 4CL, HCT, REF1, and F5H were significantly decreased in D3 compared to H3, which contrasted with the significantly increasing trend of DAMs involved in monolignol biosynthesis (**Figure 7B, E**). Overall, these findings indicated that the monolignol biosynthesis pathway genes were activated in diseased *P. notoginseng* at different ages compared to their respective healthy counterparts. However, the expression levels of DEGs and DAMs differ among healthy and diseased *P. notoginseng* at three ages, suggesting that the response mechanisms of *P. notoginseng* to RKN damage vary with age.

### 3.11 Metabolic profiling of flavonoid biosynthesis pathway in the roots of *P. notoginseng* under RKN infection

**Figure 8:**
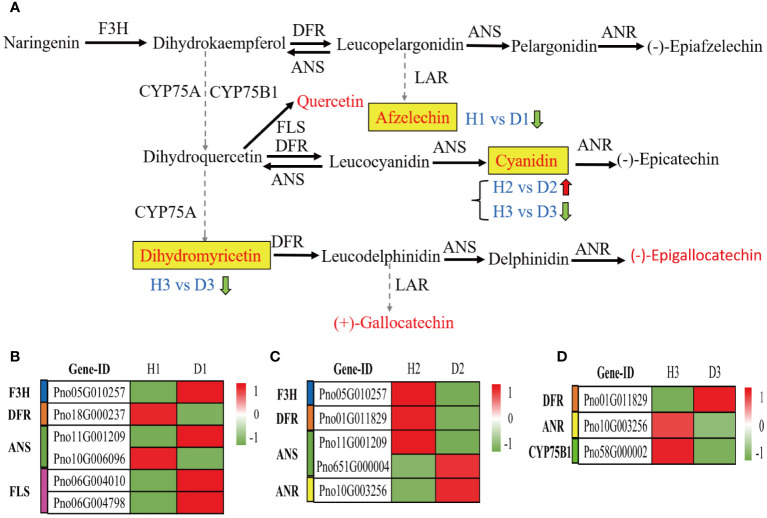
Metabolic profiling of the flavonoid biosynthesis pathway in the roots of *P. notoginseng* under RKN infection. **(A)** The key pathway of flavonoid biosynthesis (M00138). Flavonoid biosynthesis-related DEGs in H1 vs. D1 **(B)**, H2 vs. D2 **(C)**, and H3 vs. D3 **(D)**.

Further analysis of the alterations in genetic and metabolic components implicated in the flavonoid biosynthesis pathway (ko00941) within the roots of *P. notoginseng* under infection by RKN has yielded intriguing findings. Within this pathway, a total of 12 metabolites were identified, with 6 metabolites specifically associated with the crucial pathway of flavonoid biosynthesis (M00138) (**Figure 8A**). Among these metabolites, only one compound, Afzelechin (C09320), indicated a significant down-regulation in the comparison between H1 and D1 (**Figure 8A**). Notably, Cyanidin (C05905) exhibited a noteworthy up-regulation in the H2 vs. D2 comparison (**Figure 8A**). However, both cyanidin (C05905) and dihydromyricetin (C02906) showed a considerable down-regulation in the D3 relative to H3 (**Figure 8A**). Upon examining the H1 vs. D1 comparison through heatmap analysis, it was revealed that the expression of naringenin 3-dioxygenase (F3H) and flavonol synthase (FLS) genes experienced a significant up-regulation, whereas the expression of bifunctional dihydroflavonol 4-reductase (DFR) gene exhibited a noteworthy down-regulation (**Figure 8B**). Conversely, in the D2 compared to H2, the F3H and DFR gene expressions demonstrated a considerable downregulation, while the anthocyanidin reductase (ANR) gene expression exhibited a significant up-regulation (**Figure 8C**). Moreover, in the D3 compared to H3, the DFR gene expression displayed a notable up-regulation, while the expressions of flavonoid 3’-monooxygenase (CYP75B1) and ANR genes experienced a significant down-regulation (**Figure 8D**). Overall, these findings indicated the potential significance of cyanidin (C05905) in the response of two-year-old *P. notoginseng* to infestation by RKN.

### 3.12 Effect of RKN on the lignification extent in the *P. notoginseng*

**Figure 9:**
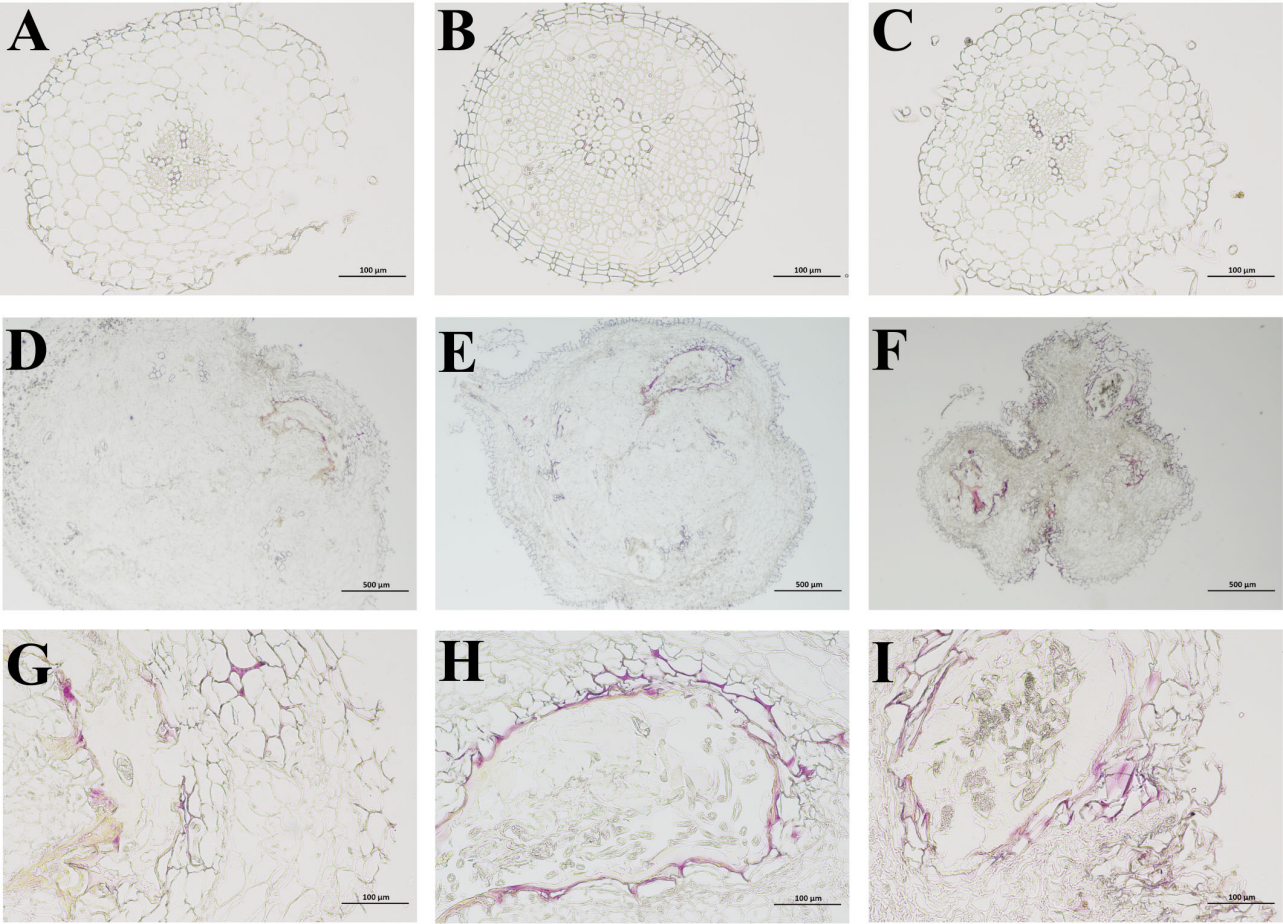
**(A–C)** represent phloroglucinol dyeing of fibrous roots in healthy one-year-old, two-year-old, and three-year-old *P. notoginseng*, respectively. **(D–F)** represent phloroglucinol dyeing of root knots in diseased one-year-old, two-year-old, and three-year-old *P. notoginseng*, respectively. **(G–I)** represent the local observation of phloroglucinol dyeing in diseased one-year-old, two-year-old, and three-year-old *P. notoginseng* root knots, respectively.

To assess the impact of RKN on lignification in *P. notoginseng* at various developmental stages, we employed the phloroglucinol hydrochloric acid (Wiesner) reaction to examine the extent of lignification in cross sections of fibrous roots and root knots, both in healthy and diseased *P. notoginseng*. In healthy *P. notoginseng*, the cross-sectional morphology of fibrous roots exhibited regularity at different ages, with neatly arranged cells and an absence of extensive cell collapse. No evident red lignified sites were observed (**Figures 9A–C**). However, in diseased *P. notoginseng*, the cross-sectional morphology of root knots indicated irregularity at different ages, with cells stacked together and intercellular tissue collapse resulting in the formation of numerous fracture cavities (**Figure 9D, E, F**). Furthermore, the presence of lignified components was indicated by a red stain, and the intercellular tissue surrounding the fracture cavities in diseased *P. notoginseng* of different ages exhibited a red coloration (**Figures 9G–H**). These findings suggested that *P. notoginseng* undergoes lignification at various developmental stages in response to RKN damage.

## 4 Discussion

### 4.1 Adult-plant resistance to RKN disease in *P. notoginseng*

Plants are vulnerable to pathogen attack during the seedling stage, but adult plants exhibit the ability to impede the infection, growth, and reproduction of the pathogen. This phenomenon has been designated as APR (Bariana and McIntosh, 1995; Wang et al., 2005). Recent investigations have revealed the existence of APR in numerous plant species, enabling them to resist various diseases. For instance, researchers have reported APR to leaf rust (Pathan and Park, 2006), stripe rust (Hiebert et al., 2010), and powdery mildew (Li et al., 2014) in different cultivars of wheat. Moreover, several APR genes have been identified in wheat and utilized in breeding programs to confer a high level of resistance (Singh et al., 2011; Agenbag et al., 2012). In our research, we observed a significant reduction in the incidence and disease index of RKN disease with the increasing age of *P. notoginseng* (**Figure 1G, H**). These findings aligned with our previous field survey, which indicated severe RKN disease in one-year-old *P. notoginseng* but only mild RKN disease in two- or three-year-old *P. notoginseng* (Wang et al., 2021). Furthermore, our other study discovered that the incidence of RKN disease in slightly infected *P. notoginseng* (level 1) was notably diminished in the following year, implying that two-year-old *P. notoginseng* demonstrated superior resistance to RKN compared to one-year-old *P. notoginseng* (Wang et al., 2023). Consequently, these results provide evidence that *P. notoginseng* possessed APR against RKN disease. However, the underlying mechanism of APR in *P. notoginseng* warrants further investigation.

### 4.2 Transcriptomic changes in healthy/diseased *P. notoginseng* at different ages

The plant-pathogen response involves the synthesis of diverse metabolites and the profound modulation of multiple genes, ultimately triggering the activation of intricate metabolic pathways and deterring pathogen infections (Wei et al., 2021; Yan et al., 2022). Comparative transcriptomics has recently gained widespread adoption for the identification of DEGs in response to various abiotic and biotic stresses (Martin et al., 2013; Zuluaga et al., 2015; Xing et al., 2017), and it has proven successful in studying the interactions between nematodes and plants such as alfalfa and rice (Haegeman et al., 2013; Postnikova et al., 2015). This study explored the impact of RKN infestation on the transcriptome and metabolome of *P. notoginseng* at different growth stages. Our transcriptome analysis revealed distinct numbers of DEGs between healthy and diseased *P. notoginseng* plants of each age group. Notably, the highest number of DEGs was observed in the comparison between two-year-old healthy and diseased plants (H2 vs. D2), with up-regulated DEGs outnumbering the down-regulated ones (**Figure 2**). These findings suggested that RKN infestation induced complex and contrasting transcriptional regulations in two-year-old *P. notoginseng*. In addition, the three comparison groups shared significant enrichment pathways, including Metabolic pathways (ko01100), Biosynthesis of secondary metabolites (ko01110), and Phenylpropanoid biosynthesis (ko00940) (**Figure 3**). Previous studies have reported the activation of the phenylpropanoid pathway or its derived molecules in response to pathogen infections in soybean or pea (Bauters et al., 2021). Moreover, luteolin, a compound known to activate the phenylpropanoid metabolic pathway, has been shown to enhance disease resistance and maintain the quality of sweet cherry (Liu et al., 2020d). Furthermore, previous research has demonstrated that the COS-OGA-induced systemic defense response in rice against RKN depended on the activation of the phenylpropanoid pathway (Singh et al., 2019). Overall, the aforementioned analyses suggested that genes related to phenylpropanoid biosynthesis may play pivotal roles in the resistance response to RKN in *P. notoginseng.*

### 4.3 Metabolome changes in healthy/diseased *P. notoginseng* at different ages

The invasion of pathogens into host plants can result in a series of metabolic changes, triggering the production of numerous secondary metabolites as a defensive response (Piasecka et al., 2015; Wei et al., 2021; Yan et al., 2022). In this study, we observed that infection by RKN induces alterations in the metabolic profile of *P. notoginseng* at different growth stages, thereby regulating the resistance of these plants to RKN infestation (**Figure 5**, **Supplementary Figure 1**). Notably, among the top three categories of DAMs, phenolic acids and flavonoids were consistently present in all three comparison groups, while lipids were found in the H2 vs. D2 and H3 vs. D3 comparison groups (**Figure 6**). This observation highlighted the pivotal roles of phenolic acids, flavonoids, and lipids in the response of *P. notoginseng* to RKN infection. Further analysis revealed a significant upregulation of lipids across all three comparison groups, and a notable up-regulation of amino acids and derivatives in both the H2 vs. D2 and H3 vs. D3 groups (**Figure 6**, **Supplementary Tables 5, 6, 7**). Moreover, an increase in phenolic acids was predominantly observed in the H1 vs. D1 group, whereas a significant downregulation of phenolic acids was noted in the H2 vs. D2 group (**Figure 6**, **Supplementary Tables 5, 7**). Previous studies have shown that phenolic acids exhibit synergistic antimicrobial activity in maize (Zhang et al., 2020a). Among these phenolic acid compounds, cinnamic acid or p-coumaric acid have been identified as potent antimicrobial agents against plant pathogens (Kim et al., 2004; Hao et al., 2010). Similarly, flavonoids played significant roles in plant defense mechanisms because of their antioxidant activities (Ahuja et al., 2012; Yadav et al., 2020; Bai et al., 2021). The KEGG enrichment analysis conducted in this investigation revealed that the DAMs in the three comparison groups were predominantly enriched in the phenylpropanoid biosynthesis (ko00940) and flavonoid biosynthesis (ko00941) (**Supplementary Figure 1**). These results aligned with previous studies indicating that plants can activate the phenylpropanoid biosynthesis and flavonoid biosynthesis pathways in response to pathogen infection. This resulted in the production of various compounds such as phenolics, flavonoids, lignin, and anthocyanin pigments (Chen et al., 2019; 2020; Mei et al., 2020; Sudheeran et al., 2021). However, the specific alterations in genes and metabolites in the phenylpropanoid biosynthesis and flavonoid biosynthesis pathways in different age groups of *P. notoginseng* under RKN infection remain unclear, and further investigation is required to identify the key genes and metabolites associated with the defense against RKN damage.

### 4.4 RKN resistance in different ages *P. notoginseng* is mediated mainly by phenylpropanoid and flavonoid biosynthesis pathways and eliciting lignification

Previous studies have indicated that the phenylpropanoid biosynthesis pathway plays a pivotal role in plant defense responses against both abiotic and biotic stresses, which can be mainly through synthesizing, various compounds, including flavonoid, lignin, hydroxycinnamic acid, coumarin, and stilbenes, to enhance the plants’ resistance to stressors (Yu and Jez, 2008; You et al., 2020; Sun et al., 2021; Zhou et al., 2021). In this study, we observed that different age groups of *P. notoginseng* activated the monolignol and flavonoid biosynthesis pathways in response to RKN infection (**Figures 7** , **8**). Specifically, in comparison to one-year-old healthy *P. notoginseng*, the content of 5-O-p-coumaroylquinic acid and the expression levels of PAL, CCR, CYP73A, CYP98A, F3H, and FLS genes were significantly up-regulated. Additionally, most genes involved in POD and CAD exhibited an overall upregulation trend in one-year-old diseased *P. notoginseng* (**Figures 7** , **8**). PAL, a rate-limiting enzyme in the phenylpropanoid pathway, indirectly contributes to monolignol biosynthesis (Dixon et al., 2002; Zhang and Liu, 2015; Zhou et al., 2021). Furthermore, PAL and POD are crucial enzymes involved in the resistance of host plants against pest insects and pathogens (Han et al., 2009). Our results revealed that one-year-old *P. notoginseng* responded to RKN infection by inducing higher expression of PAL (**Figure 7C**), aligning with certain previous studies (Chaman et al, 2003; Han et al., 2009; Mei et al., 2020). In addition, CAD and CCR have been recognized as pivotal enzymatic steps in monolignol biosynthesis (Vogt, 2010). It has been reported that the damage to the flavedo by oleocellosis led to increased expression of CCR (Zhou et al., 2021). Similarly, our results revealed higher expression of CCR in one-year-old diseased *P. notoginseng* (**Figure 7C**). Collectively, these findings suggested that in response to RKN infection, one-year-old *P. notoginseng* may up-regulate the production of 5-O-p-Coumaroylquinic acid and key genes involved in monolignol biosynthesis, thereby activating the plant defense system.

Numerous prior studies have provided evidence regarding the contributions of chlorogenic acid, quercetin, and kaempferol to plant defense responses against fungal and bacterial infections (Takahama and Hirota, 2000; Rao et al., 2007; Martinez et al., 2017). In our study, we observed significant downregulation in the content of chlorogenic acid and 1-O-sinapoyl-D-glucose, as well as the expression levels of PAL, 4CL, CYP73A, CCoAOMT, F5H, COMT, F3H, and DFR genes in two-year-old diseased *P. notoginseng*, when compared to two-year-old healthy plants (**Figures 7**,**8**). Previous studies have reported the antifungal activity of chlorogenic acid and its derivatives (Ma et al., 2007; Sung and Lee, 2010), with hydrolysis of chlorogenic acid into 4-hydroxybenzoic acid being implicated in increasing apple resistance against fungal infection (Fawcett and Spencer, 1968). In addition, a study revealed a negative correlation between chlorogenic acid and root gall index in tomato roots, suggesting its role in RKN resistance (Rani et al., 2008). Thus, we inferred that chlorogenic acid may play a crucial role in the response of two-year-old *P. notoginseng* to RKN attack. Remarkably, the content of cyanidin and the expression levels of ANR were upregulated in the two-year-old diseased *P. notoginseng* compared to healthy plants (**Figure 8**). Moreover, cyanidin served as a precursor to epicatechin, ANR was an essential enzyme specifically responsible for the final step in the synthesis of epicatechin (**Figure 8**), which influenced the content of epicatechin. Previous studies have demonstrated that epicatechin contributed to pathogen resistance by inhibiting the activities of pectate lyase (PL), polygalacturonase (PG) (Prusky et al., 1988; Zhang et al., 2020b). Therefore, we suggested that two-year-old *P. notoginseng* may exhibit resistance to nematode attacks by consuming compounds such as chlorogenic acid in the phenylpropanoid pathway. Meanwhile, it may significantly increase the content of cyanidin and the expression of ANR in the flavonoid pathway to indirectly enhance resistance against nematode infection.

Recent studies have provided compelling evidence highlighting the role of lignification in the defense mechanisms against both biotic and abiotic stresses (Cabane et al., 2012; Cesarino, 2019; Zhou et al., 2021). In our study, a considerable number of DEGs and metabolites in *P. notoginseng* were associated with monolignol biosynthesis (**Figure 7**). Our findings revealed that p-coumaric acid, chlorogenic acid, 1-O-sinapoyl-D-glucose, coniferyl alcohol, ferulic acid, and sinapyl alcohol exhibited increased levels in D3 compared to H3, whereas most DEGs indicated significant down-regulation (**Figures 7B, E**). This observation suggested that RKN infection may stimulate the synthesis of phenolic acids at a faster rate than the decrease in the activity of many enzymes in *P. notoginseng*. A previous study reported an increase in lignin and polyphenols in the flavedo damaged by oleocellosis (Zhou et al., 2021), as well as the higher levels of p-coumaric acid and ferulic acid in wheat seeds than that in control seed when infested by midge larvae (Ding et al., 2000). Moreover, transgenic tomato plants with increased levels of chlorogenic acid exhibited enhanced resistance against the bacterial pathogen *Pseudomonas syringae* (Niggeweg et al., 2004). Therefore, our results suggested that three-year-old *P. notoginseng* enhanced their resistance to nematode infection by producing significant amounts of phenolic acids involved in monolignol biosynthesis. Therefore, we observed lignification in the intercellular tissues surrounding the fracture cavities in different age groups of diseased *P. notoginseng* (**Figures 8G–I**). These findings aligned with previous studies demonstrating that lignin-deposited structures served as physical barriers, and the lignification of cell walls spatially restricted the spread and growth of invading pathogens (Lee et al., 2018; Lee et al., 2019; Zhou et al., 2021). Combining these results with the activation of monolignol and flavonoid biosynthesis pathways in response to RKN infection in different age groups of *P. notoginseng*, we concluded that all age groups of *P. notoginseng* employed the accumulation or utilization of resistance-related compounds and induce tissue cell lignification to prevent further spread and growth of RKN.

### 4.5 Metabolites and genes in the phenylpropane and flavonoid biosynthesis pathways play a crucial role in APR to *P. notoginseng* RKN disease

Previous studies have shown that a combination of APR genes can improve crop resistance, which is deemed an effective management strategy (Singh et al., 2011). In this study, we found that all different age groups of *P. notoginseng* can activate the phenylpropanoid biosynthesis and flavonoid biosynthesis pathways in response to RKN infection. However, it is unclear which metabolites and genes in the phenylpropane and flavonoid biosynthesis pathways are involved in APR to *P. notoginseng* RKN disease. Hence, we analyzed the correlation between all DAMs/DEGs in these two pathways and the disease incidence/index of RKN disease in different ages *P. notoginseng*. Our findings revealed that a total of eight compounds in the phenylpropane and flavonoid biosynthesis pathways, namely, p-coumaric acid, sinapinaldehyd, 1-O-sinapoyl-D-glucose, sinapyl alcohol, 2-hydroxycinnamic acid, cyanidin, gallocatechin, and phloretin-2’-O-glucoside exhibited significantly negatively correlate with the disease incidence/index of RKN disease in different ages *P. notoginseng* (**Supplementary Table 8**). In addition, the phenylpropane and flavonoid biosynthesis pathways, including CAD (12 genes) and POD (7 genes) are involved in the biosynthetic pathways of phenylpropane and flavonoids, β- glucosidase (6 genes), HCT (5 genes), COMT (3 genes), CCR (3 genes), CCoAOMT (2 genes), 4CL (2 genes), CYP73A (1 gene), CYP98A (1 gene), FLS (1 gene), and chalcone isomerase (CHI) (1 gene) were significantly negatively correlated with the disease incidence/index of RKN disease in different ages *P. notoginseng* (**Supplementary Table 9**). Previous studies have indicated that defense genes of PAL, LOX, and PBZ1 are all involved in APR to rice bacterial blight, and these genes were induced by pathogen both in seedling and adult plants, and it was stronger in adult plants than that in seedlings (Sha et al., 2005). Thus, we inferred that p-coumaric acid, sinapinaldehyd, 1-O-sinapoyl-D-glucose, sinapyl alcohol, 2-hydroxycinnamic acid, cyanidin, gallocatechin, phloretin-2’-O-glucoside compounds and CAD, POD, β-glucosidase, HCT, COMT, CCR, CCoAOMT, 4CL, CYP73A, CYP98A, FLS, CHI genes are all involved in APR to RKN disease in *P. notoginseng*, these compounds and genes may play a crucial role in APR to RKN disease. In this study, the results can provide new resources for the prevention and sustainable control of RKN disease in *P. notoginseng*, and provide an important theoretical basis for further clarifying the mechanism of different ages *P. notoginseng* to RKN infection. At present, there is still very little research on the APR genes and metabolites of *P. notoginseng* to RKN disease, the real role and mode of action of the APR genes and metabolites need to further study.

## 5 Conclusions

In this study, we employed transcriptome, metabolome, and histochemistry analyses to explore the DEGs, DAMs, and lignin accumulation in response to RKN infection across different age groups of *P. notoginseng*. Our findings revealed that the various age groups of *P. notoginseng* exhibited distinct modes of activation within the phenylpropanoid and flavonoid biosynthesis pathways in response to RKN infection. One-year-old *P. notoginseng* appeared to resist RKN attack by up-regulating the expression of key genes involved in monolignol biosynthesis, along with an increase in 5-O-p-coumaroylquinic acid. Two-year-old *P. notoginseng* promoted the resistance by depleting chlorogenic acid levels and increasing cyanidin content. Three-year-old *P. notoginseng* enhanced its resistance through a significant elevation in the levels of five phenolic acids associated with monolignol biosynthesis. Notably, we observed that *P. notoginseng* can establish a lignin barrier, restricting the spread and growth of pathogens to the site of infection. In summary, *P. notoginseng* employed a multifaceted defense strategy to prevent further spread and growth of RKN. This included the accumulation or depletion of resistance-related compounds involved in the phenylpropanoid and flavonoid pathways, as well as the induction of lignification in tissue cells.

## Data availability statement

The datasets presented in this study can be found in online repositories. The names of the repository/repositories and accession number(s) can be found below: https://www.ncbi.nlm.nih.gov/,PRJNA983978.

## Author contributions

ZHW: Data curation, Investigation, Validation, Writing –original draft, Writing – review & editing. WPW: Funding acquisition, Investigation, Writing – original draft. WTW: Data curation, Investigation, Writing – review & editing. HW: Data curation, Writing – review & editing. SZ: Data curation, Investigation, Writing – review & editing. CY: Supervision, Writing – review & editing. LG: Supervision, Writing – review & editing. ZXW: Supervision, Writing – review & editing. HH:Supervision, Writing – review & editing. YL: Supervision, Writing –review & editing. SSZ: Funding acquisition, Supervision, Writing –review & editing. YZ: Supervision, Writing – review & editing. YW: Funding acquisition, Supervision, Writing – review & editing. XH: Funding acquisition, Supervision, Writing – review & editing.
