# Supplementary material for: Integrated analysis of transcriptome, metabolome, and histochemistry reveals the response mechanisms of different ages Panax notoginseng to root-knot nematode infection
Source: Front Plant Sci. 2023 Sep 14;14:1258316. doi: 10.3389/fpls.2023.1258316 (PMC10539906; doi:10.3389/fpls.2023.1258316)
Supplement: Supplementary file 1 [file DataSheet_1.docx]

***Supplementary Material***


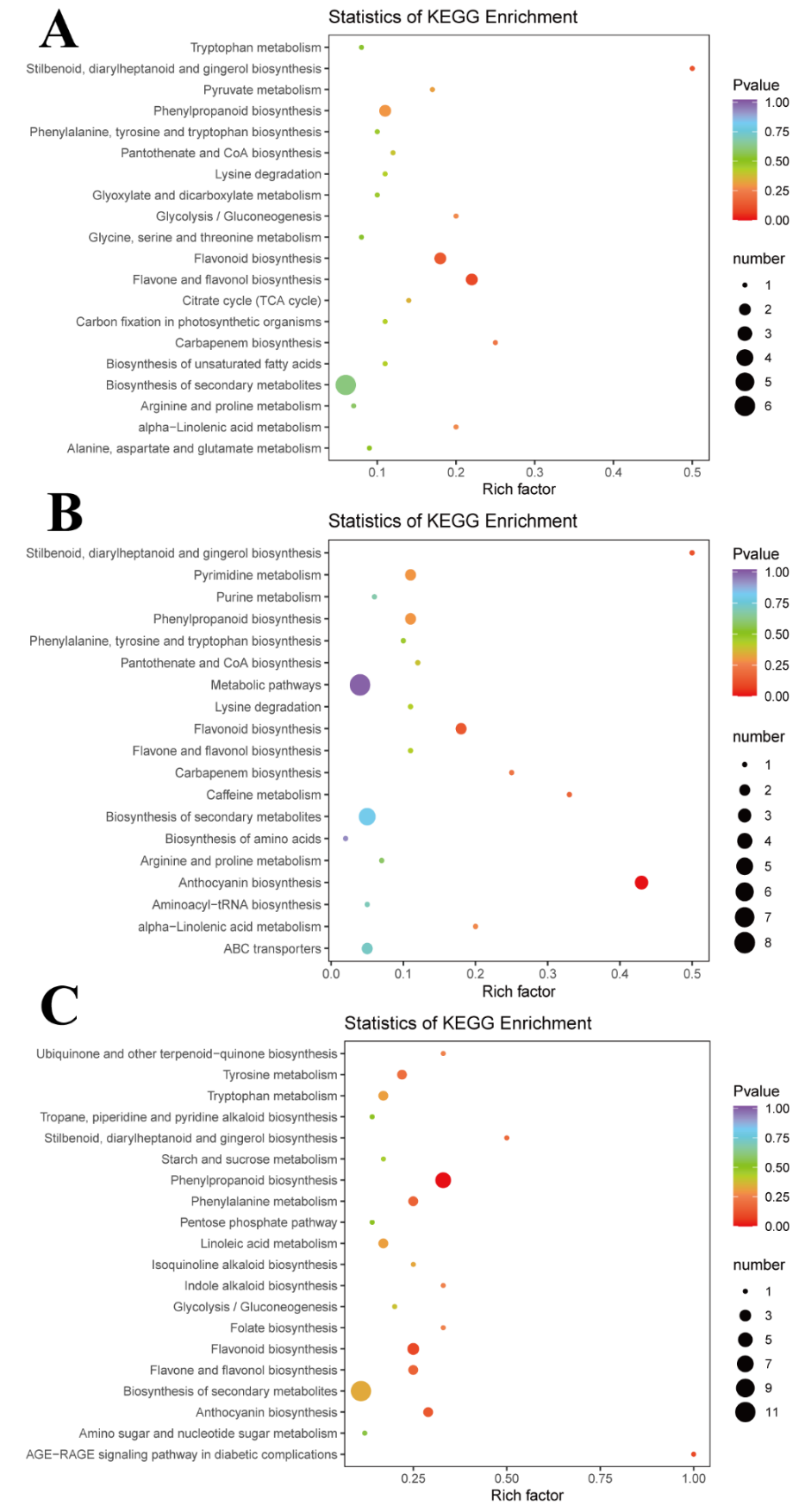


**Supplementary Figure 1.** KEGG pathway enrichment analysis of differentially accumulated metabolites (DAMs) of H1 vs. D1 **(A)**, H2 vs. D2 **(B)**, H3 vs. D3 **(C)**. The color of the point represents p, and the size of the point represents the number of differentially enriched metabolites.


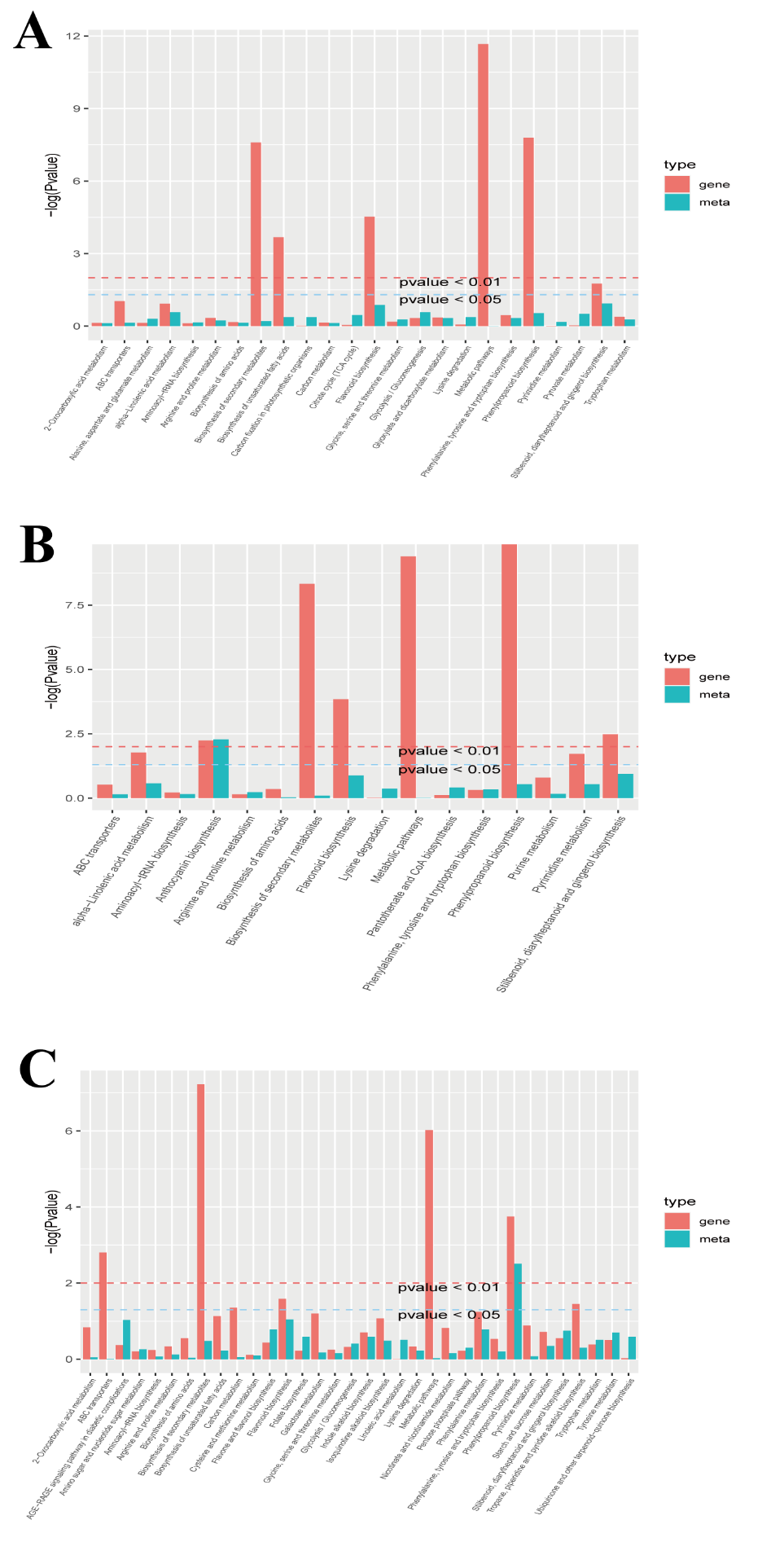


**Supplementary Figure 2.** Joint KEGG enrichment P-value histogram of H1 vs. D1 **(A)**, H2 vs. D2 **(B)**, and H3 vs. D3 **(C)**.
